# Supplementary material for: A Critical View of the Application of the APEX Software (Aqueous Photochemistry of Environmentally-Occurring Xenobiotics) to Predict Photoreaction Kinetics in Surface Freshwaters
Source: Molecules. 2019 Dec 18;25(1):9. doi: 10.3390/molecules25010009 (PMC7017383; doi:10.3390/molecules25010009)
Supplement: Supplementary file 1 [file molecules-25-00009-s001.zip › 3.2.4_gcc-4.4.0.zip!/3.2.4_gcc-4.4.0/3.2.4_gcc-4.4.0/doc/octave/html/interpreter/Leaving-Debug-Mode.html]

Leaving Debug Mode - Untitled


Next: Breakpoints,
Previous: Entering Debug Mode,
Up: Debugging


---

### 13.2 Leavinging Debug Mode

To leave the debug mode, use either `dbcont`
or `return`.

— Command: **dbcont** ()  
> In debugging mode, quit debugging mode and continue execution.
>
>
>
> **See also:** dbstep, dbstep.

To quit debug mode and return directly to the prompt `dbquit`
should be used instead

— Command: **dbquit** ()  
> In debugging mode, quit debugging mode and return to the top level.
>
>
>
> **See also:** dbstep, dbcont.

Finally, typing `exit` or `quit` at the debug prompt will
result in Octave terminating normally.
